# Healthcare workers’ knowledge, preparedness, counselling practices, and perceived barriers to confront COVID-19: A cross-sectional study from a war-torn country, Yemen

**DOI:** 10.1371/journal.pone.0243962

**Published:** 2020-12-11

**Authors:** Fahmi Y. Al-Ashwal, Mohammed Kubas, Mohammed Zawiah, Ahmad Naoras Bitar, Ramzi Mukred Saeed, Syed Azhar Syed Sulaiman, Amer Hayat Khan, Siti Maisharah Sheikh Ghadzi

**Affiliations:** 1 Clinical Pharmacy Department, University of Science and Technology Hospital (USTH), Sana'a, Yemen; 2 Discipline of Clinical Pharmacy, School of Pharmaceutical Sciences, Universiti Sains Malaysia, Penang, Malaysia; 3 Pharmacy Practice Department, Kulliyyah of Pharmacy, International Islamic University Malaysia (IIUM), Kuantan, Pahang, Malaysia; 4 Department of Pharmacy Practice, College of Clinical Pharmacy, University of Al Hodeida, Al Hodeida, Yemen; 5 Department of Clinical Pharmacy and Pharmacy Practice, University of Science and Technology (UST), Sana'a, Yemen; 6 Department of Pharmaceutical sciences, School of Pharmacy, The University of Jordan, Amman, Jordan; 7 Advanced Medical and Dental Institute, Universiti Sains Malaysia, Kepala Batas, Penang, Malaysia; Waikato Institute of Technology, NEW ZEALAND

## Abstract

**Background:**

The coronavirus disease of 2019 (COVID-19) represents a difficult challenge and could have devastating consequences for the healthcare system and healthcare workers in war-torn countries with poor healthcare facilities such as Yemen. Our study aimed to evaluate the knowledge, preparedness, counselling practices of healthcare workers regarding COVID-19, and the perceived barriers to adequately prevent and control COVID-19 in Yemen.

**Methods:**

Healthcare workers (HCWs) from major healthcare facilities participated in this cross-sectional study. A self-administered questionnaire comprising of five main domains (demographics, knowledge, self-preparedness, counselling practice, perceived barriers) was distributed among HCWs after obtaining informed consent. A convenient sampling technique was used. Descriptive and inferential analyses were applied using SPSS software.

**Results:**

A total of 1000 participants were initially targeted to participate in the study with 514 (51.4%) responding, of which 55.3% were female. Physicians and nurses constituted the largest proportion of participants, with 39.5% and 33.3%, respectively. The median scores for knowledge, self-preparedness, and counselling practice were 8 (out of 9), 9 (out of 15), and 25 (out of 30), respectively. The physician group showed a statistically significant association with better knowledge compared to the nurse group only, P<0.001. Males had higher preparedness scores than females, p<0.001. Also, the intensive care unit (ICU) and emergency departments presented a statistically significant difference by which the participants from these departments were more prepared compared to the others (e.g. outpatients, paediatrics and surgery) with P < 0.0001. The lack of awareness among the general population about COVID-19 preventive measures was perceived as the most common barrier for the adequate prevention and control of COVID-19 in Yemen (89.1%).

**Conclusion:**

The major highlight of this study is that HCWs have, overall, good knowledge, suboptimal preparedness, and adequate counselling practices prior to the outbreak of COVID-19 in Yemen, despite the high number of perceived barriers. However, urgent action and interventions are needed to improve the preparedness of HCWs to manage COVID-19. The perceived barriers also need to be fully addressed by the local healthcare authorities and international organisations working in Yemen for adequate prevention and control measures to be in place in managing COVID-19.

## Introduction

The coronavirus disease of 2019 (COVID-19) is a newly emerging infectious disease and a rapidly expanding pandemic caused by a novel human coronavirus called severe acute respiratory syndrome coronavirus 2 (SARS-COV-2), first detected in Wuhan, China in December 2019 [1]. On March 11, 2020, and after the rapid spread and continuous increase in the number of cases, deaths, and countries affected by the disease outbreak, the World Health Organization (WHO) declared the COVID-19 a global pandemic [2]. As of July 13, 2020, more than 12,700,000 cases have been reported globally, with approximately 566,654 deaths [3]. In Yemen, the first confirmed case of COVID-19 was announced on April 10, 2020 [4]. Currently, as of July 13, 2020, approximately 1469 cases of COVID-19 have been officially confirmed with 418 deaths, and the ratio of the total reported deaths to the total reported confirmed cases was the highest among all countries [3]. This could be due to the severe shortage of testing laboratories and lack of essential resources for the management of severe cases of COVID-19 in the majority of the healthcare facilities [5, 6]. In addition, the surveillance system in Yemen was only able to capture severe cases of infection as individuals with mild to moderate infection of the disease were not self-reported to healthcare facilities [7].

The healthcare system in Yemen is seriously underdeveloped with many weaknesses and challenges, including insufficient and partially inadequate healthcare facilities, shortage of healthcare workers (HCWs), lack of essential medical equipment, and unequal distribution of human and medical resources [8, 9]. After more than five years of conflict and war, these challenges have amplified, and the healthcare infrastructure has been heavily devastated, with only 51% of healthcare facilities are fully functioning [10]. Furthermore, there are only 10 HCWs per 10000 people in Yemen, which is less than half the WHO benchmark (> 22 HCW per 10000 people), with no medical specialists in 38% of hospitals and no doctors in 18% of districts. Moreover, only 1 out of 22 governorates has adequate inpatient beds according to the WHO minimum benchmark of more than 10 beds per 10000 people [11]. The United Nations (UN) agencies and international non-government organisations (NGOs) have expressed concern that Yemen’s healthcare system is collapsing and facing a major COVID‐19 crisis [12]. Also, considering that a large number of COVID-19 cases are mild or asymptomatic, and with testing kits in short supply with testing performed only on a small percentage of the population, the number of actual cases and deaths in Yemen is expected to be several multiples higher than the official reported figures [13]. Therefore, the implementation of preventive measures is essential to mitigate against the uncontrolled spread of the virus.

Currently, there is no specific nor recognised treatment or effective vaccine available for COVID-19 [14], and non-pharmacological interventions are being the most effective methods to mitigate the spread of COVID-19 [15]. As such, HCWs play a critical role in the prevention and control of COVID-19 [16]. Their counselling practices are of vital importance to improve the awareness of patients and the community about COVID-19 preventive measures such as social distancing, use of face masks and washing hands. Also, their knowledge and preparedness in the management of COVID-19 are important to prevent and control the spread of this infectious disease [17]. In Yemen, good knowledge and adequate preparedness of HCWs are extra important because ‎these skills need to compensate, even partially, for the already inefficient healthcare system. Therefore, as the threat of COVID-19 grows, HCW’s knowledge, preparedness, and the practice of patient counselling about COVID-19 need to be ascertained.

Several studies have been conducted globally to evaluate both the knowledge and preparedness of HCW’s in combatting COVID-19 [18–21]. However, to the best of our knowledge, this is the first paper-based study conducted in a Middle Eastern country prior to its healthcare authorities announcing their first confirmed case of COVID-19. Also, no previous studies in Yemen have evaluated the self-preparedness of frontline HCWs towards COVID-19, and likewise, no previous research has been undertaken to investigate the practices of HCWs and patient counselling concerning COVID-19 preventive measures. Therefore, this study aims to assess the knowledge, preparedness, and counselling practices of HCWs in relation to COVID-19. Also, to address the potential barriers and challenges for adequate prevention and control of COVID-19 in Yemen.

## Materials and methods

### Study design and setting

A cross-sectional study was conducted over three weeks between March 24, 2020, and April 14, 2020, among HCWs in Sana’a, the capital of Yemen. This survey was undertaken before the health authorities in Sana'a announced the first confirmed case of COVID-19. Seven research assistants distributed the questionnaires in 11 major healthcare facilities in Sana’a city (seven private hospitals, two government hospitals, and two primary care centres). The population of the study included physicians, nurses, pharmacists, and assistant physicians working in these healthcare facilities. A convenient sampling method was used to select the participants.

### Data collection tool

A self-administered structural questionnaire was developed, and adapted from previously published studies as guidance [22, 23]. Four independent healthcare professionals evaluated the initial draft of the questionnaire for face validity, in which changes were made according to their feedback and comments. The final form of the questionnaire consisted of five sections (S1 File). The first section addressed the demographic data such as age, gender, specialty (physician, nurse, pharmacist, and assistant physician), department (intensive care unit (ICU), emergency, internal medicine, and others), type of healthcare facility (government, private and healthcare centres), and experience years. This section also included general questions relating to the sources of information about COVID-19 and whether they had been trained on prevention and control measures for handling COVID-19 or other infectious diseases. Section two included nine questions exploring the knowledge of HCWs about COVID-19 pandemic, provided with "yes”; “no”; or “I don't know" choices. Questions in this section assessed the information of the respondents about the virus, common symptoms of the disease, people who are more vulnerable to serious illness, mode of transmission, incubation period, preventive measures, and the availability of a vaccine. Each correct answer was rated with a score of 1, and for an incorrect response (wrong answer or "do not know"), a 0 score was awarded [20, 22]. Thus, the total score for knowledge ranged between 0 and 9 points. The third section of the questionnaire contained fifteen questions with “yes”; “no”; or “not sure” choices. This section evaluated healthcare professionals’ general preparedness of COVID-19 in terms of their knowledge, level of skills to care and manage patients with COVID-19, and the ability to perform isolation and decontamination procedures for infectious diseases. Those who answered “yes” were given a 1 point score, while those not prepared or were not sure were awarded a score of 0. Accordingly, the total score for preparedness ranged between 0 and 15. Section four contained six questions on 5-point Likert scale (never “1”, rarely “2”, sometimes “3”, usually “4” and always “5”). Questions in this section evaluated healthcare professionals’ practices in educating their patients on appropriate preventive measures for COVID-19 before the outbreak occurred in Yemen, with the total ranging between 6 and 30. The last section analysed the potential barriers and challenges for the prevention and control of COVID-19.

### Ethical approval

Ethical approval for the study was obtained from the Ethical Committee of the Medical Research, University of Sciences and Technology, Sana’a, Yemen (ECA/UST189). Permission to conduct the study and distribute the questionnaires among the HCWs was granted by each respective hospital. Verbal informed consent from participants was acceptable and approved by the ethical committee. The participants were adequately informed about the study’s objectives and verbally consented to participate.

### Statistical analysis

Data was initially entered into the Microsoft Excel spreadsheet then exported into IBM statistical package for the social sciences (IBM SPSS version 23). Both descriptive and inferential statistics were utilised. Categorical variables were described using frequencies and percentages, and the median with interquartile range (IQR) were used for continuous variables. The associations between knowledge, preparedness, and practice scores and respondents’ characteristics were assessed using the nonparametric Mann-Whitney U test and Kruskal–Wallis (KW) test, with a p-value of less than 0.05considered statistically significant.

### Sample size calculation

Physicians, nurses, pharmacists, and assistant physicians working in the selected hospitals participated in the study. A sample size of 377 was estimated using the online Raosoft sample size calculator (available at http://www.raosoft.com/samplesize.html), assuming a response distribution of 50% with a 95% confidence interval and a 5% margin of error. Adding to them 20% in case there was an error in questionnaire filling or if there were responses with large missing data. Hence, the final sample size of HCWs for the study was estimated to be 471.

## Results

### The demographic data of participants

A total of 514 respondents were included in the final analysis, of which 55.3% were female. Physicians and nurses made up the largest proportion of the participants, with 39.5% and 33.3%, respectively, while pharmacists represented 10.5% only. The median for the respondents’ age was 28 years, and the experience was 5 years. The vast majority of participant were from public and private hospitals, 52.3% and 40.7%, respectively. The demographic characteristics of the participants are shown in Table 1. The participants who attended workshops or educational courses about COVID-19 represented only 26.8% of the recruited sample, while 24.7% received some professional training for COVID-19, and 56.2% had some training for controlling infectious disease outbreaks. Notably, over half of the recruited sample (53.9%) had experience in managing infectious disease outbreaks.

**Table 1:** Participants' demographics.

Variable	Number	Percentage
**Gender**		
Male	230	44.7
Female	284	55.3
**Age (years)**		
≤28	271	52.7
>28	243	47.3
**Experience (years)**		
0–2	155	30.2
3–5	190	37.0
6–8	70	13.6
≥9	99	19.3
**Profession**		
Physician	203	39.5
Nurse	171	33.3
Physician assistant	86	16.7
Pharmacist	54	10.5
**Working place**		
Governmental hospital	269	52.3
Private hospital	209	40.7
Primary healthcare centre	19	3.7
Missing	17	3.3
**Department**		
ICU	41	8.0
Emergency	64	12.5
Internal medicine	71	13.8
Others (e.g. Outpatients, pediatrics and surgery)	329	64.0
Missing	9	1.8

### Knowledge of HCWs about the COVID-19 pandemic and sources of information

Concerning their knowledge, the majority of participants demonstrated good knowledge about COVID-19 for most of the items in this section, with an overall median (IQR) score of 8 (8–9). The highest scores for knowledge were for the questions related to the symptoms of COVID-19 (95.3%), the importance of using a facemask in the prevention of the disease (95.3%), and mode of virus transmission (92.2%). Similarly, over 90% of HCWs correctly identified the individuals who are at higher risk for severe illness. However, 37% of respondents did not know that there is a chance for asymptomatic patients to transmit the disease, and only around two-thirds of HCWs (67.5%) were aware of the scientific name of the causative virus for COVID-*19* (Table 2). The physician group showed a statistically significant association with better knowledge compared to the nurse group only, P<0.001 (Table 5).

**Table 2:** Knowledge of the HCWs about the COVID-19 pandemic.

Statement	Correct answers, N(%)	Overall score, Median (IQR)
COVID-19 is an infectious disease caused by a virus called severe acute respiratory syndrome coronavirus 2 (SARS-CoV-2)	347(67.5)	
The most common symptoms of COVID-19 are fever, tiredness, shortness of breath and dry cough	490(95.3)	
Older people, and those with underlying medical problems like heart problems or diabetes, are more likely to develop serious illness	467(90.9)	8 (7–9)
The disease spread from person to person through small droplets from the nose or mouth when a person with COVID-19 coughs, sneezes or exhales	474(92.2)	
There is a chance that people may get infected from asymptomatic COVID-19 patient	326(63.4)	
Studies indicate that the virus causing COVID-19 may remain alive on surfaces for a few hours to several days	439 (85.4)	
The time between catching the virus and symptoms onset (incubation period) for COVID-19 is from 2–14 days	457 (88.9)	
The use of masks is crucial for health workers and people who are taking care of someone (at home or in a health care facility)	490 (95.3)	
Currently, there is no vaccine to prevent COVID-19 disease	440 (85.6)	

IQR = Interquartile range.

**Fig 1:**
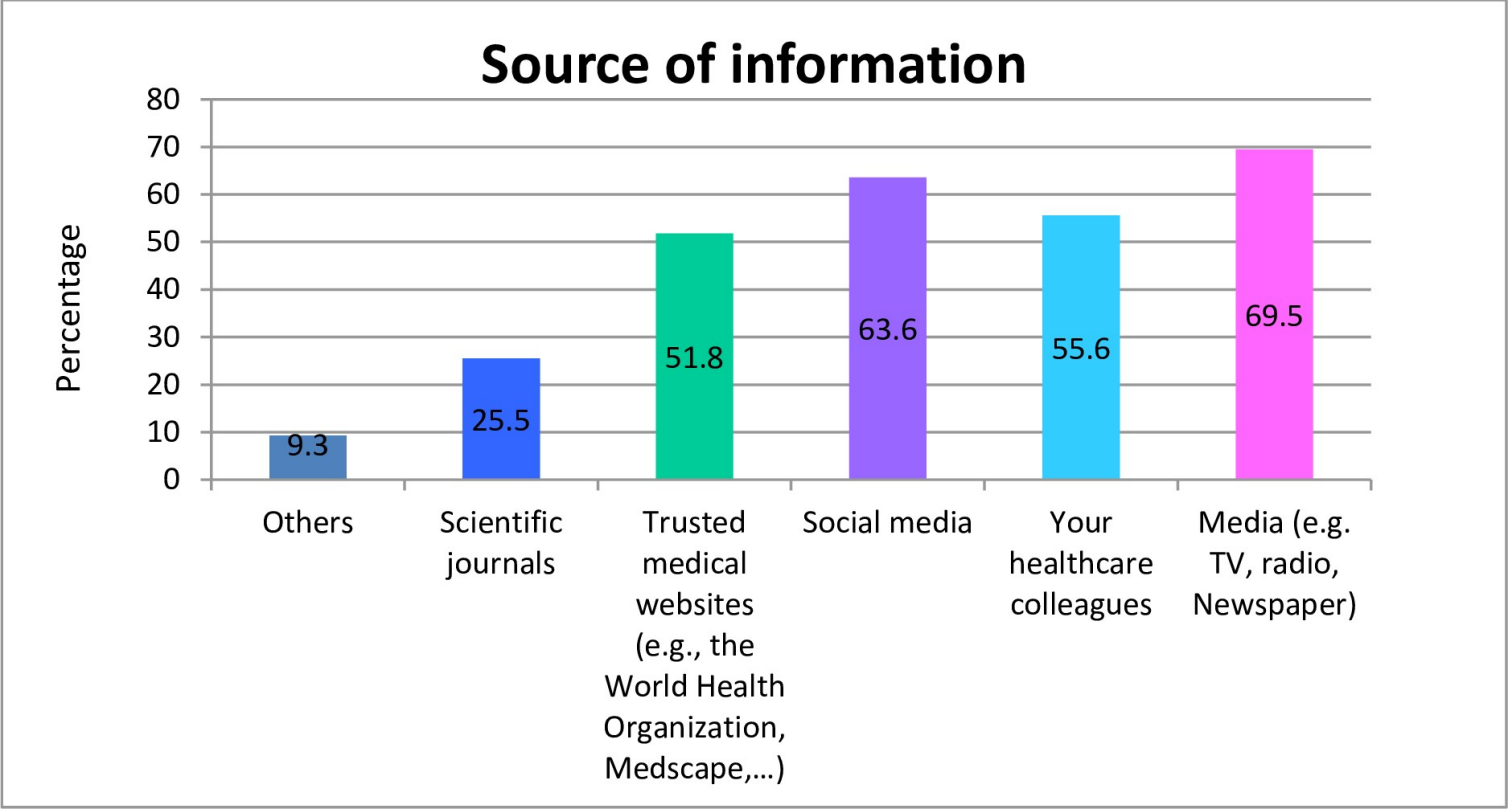
Source of information regarding COVID-19.

Fig 1 summarises the different information sources used by HCWs to obtain knowledge regarding COVID-19. The majority of the participants reported television (TV) and radio as the main source of information (69.5%), followed by social media (63.6%) such as Facebook and WhatsApp. However, only 25.5% of HCWs acquired knowledge from peer-reviewed scientific articles.

### Self-preparedness of HCWs for COVID-19

Regarding the preparedness to face COVID-19, the HCWs surveyed in this study demonstrated suboptimal preparedness levels with an overall median (IQR) score of 9 (6–11) out of 15 items. The preparedness scores fluctuated between 18% and 85% depending on the items asked (Table 3). The majority of participants (84.6%) had read general guidance about COVID-19 (e.g., WHO and CDC guidance), and approximately two-thirds (67.3%) were confident in their ability and skills to educate patients and community members about COVID-19 preventive measures. Participants who could identify the signs and symptoms of COVID-19 represented around 66%, while those who believed they had the skills to manage these symptoms and prioritise patients based on the need or situation represented 44.2% and 41.2%, respectively. However, only 94 (18%) were confident enough to take care of a COVID-19 patient without any supervision.

**Table 3:** Self-preparedness of HCWs for the COVID-19.

Statement	Answers with "Yes", N (%)	Overall score Median (IQR)
I know all the relevant information related to COVID-19 disease.	288(56.0)	
I have read general guidance about COVID-19 (for example, WHO or CDC COVID-19 guidance).	435 (84.6)	
I have read medical articles related to COVID-19 outbreak preparedness.	392 (76.3)	
I can identify the signs and symptoms of COVID-19.	342(66.5)	
I can manage the common symptoms of COVID-19	227 (44.2)	
I have the skills to decide which patients should be managed first.	212 (41.2)	9 (6–11)
I can care for COVID-19 patients independently without any supervision.	94 (18.3)	
I have participated in educational activities dealing with COVID-19 outbreak preparedness (continuing education classes, seminars, or courses).	143 (27.8)	
I have the necessary knowledge and skills to educate patients about COVID-19 prevention practices.	346 (67.3)	
I am aware of all the challenges in my community that may hinder the response to the COVID-19	331 (64.4)	
I know how and to whom COVID-19 cases should be reported.	256 (49.8)	
I have participated in emergency planning for COVID-19 in my community	107 (20.8)	
In case of infectious disease outbreaks, I know how to use properly the personal protective equipment	357 (69.5)	
In case of infectious disease outbreaks, I know how to execute decontamination procedures.	390 (75.9)	
In a case of emergency, I know how to perform isolation procedures to minimize the risks of community exposure.	349 (67.9)	

IQR = Interquartile range.

Remarkably, a significant proportion of respondents (76.3%) read medical articles related to preparedness for a COVID-19 outbreak. However, a low percentage of HCWs (27.8%) participated in educational activities (courses and seminars) about COVID-19 preparedness, with only one-fifth (20.8%) practically involved in emergency planning for COVID-19 before the outbreak. Moreover, almost 50% of respondents were uncertain or did not know to whom or where COVID-19 cases should be reported. Notably, a large proportion of HCWs stated that they knew how to execute decontamination and isolation procedures for infectious disease outbreaks with scores reported at 75.9% and 67.9%, respectively. Males had higher preparedness scores compared to females, p < 0.001. Also, the ICU and emergency departments presented a statistically significant difference concerning preparedness where participants from these departments tended to be more prepared compared to others (e.g. outpatients, paediatrics and surgery) with P < 0.0001 (Table 5).

### Counselling practices

The overall median (IQR) score for practice was 25 (21–29) out of 30. Here, over 60% of the participants reported either usually or always educating their patients about the signs and symptoms of COVID-19. Also, almost 59% reported always advising their patients to wash their hands properly before touching their faces, and 24% usually did so. Three-quarters of the respondents (75.3%) reported either always or usually discussing the importance of social distancing and avoiding public gatherings such as khat sessions, weddings, malls, markets, and restaurants with their patients. Overall, the inappropriate practices (never or seldomly counselled their patients about preventive measures) were observed in less than 10% for all questions except for the one related to patients counselling on what to do if they developed similar symptoms and signs of COVID-19, which was slightly higher with 15.1% (Table 4). There was no statistically significant association between the overall score for counselling practices and demographic variables (Table 5).

**Table 4:** Counselling practices.

Statement	Always N (%)	Usually N (%)	Sometimes N (%)	Rarely N (%)	Never N (%)	Overall score, Median (IQR)
Do you educate the patients and the people around you about the risk of COVID-19?	170 (33.1)	172 (33.5)	135 (26.3)	22 (4.3)	13 (2.5)	
Do you educate your patients about the symptoms, signs, and mode of transmission of COVID-19?	185 (36.0)	143 (27.8)	135 (26.3)	34 (6.6)	16 (3.1)	25 (21–29)
Do you educate the patients and the people around you about the preventative measures of COVID-19?	198 (38.5)	154 (30.0)	111 (21.6)	35 (6.8)	16 (3.1)	
Do you advise the patients and the people around you to avoid public gatherings (such as khat sessions, weddings, malls, markets, and restaurants)	262 (50.0)	130 (25.3)	83 (16.1)	25 (4.9)	13 (2.5)	
Do you educate the patients and the people around you about the importance of handwashing before touching their eyes, nose, or mouth?	301 (58.6)	124 (24.1)	68 (13.2)	15 (2.9)	6 (1.2)	
Do you educate the patients and the people around you on what to do if they developed similar symptoms and signs to COVID-19 disease	230 (44.7)	122 (23.7)	81 (15.8)	51 (9.9)	27 (5.3)	

IQR = Interquartile range.

**Table 5:** The effect of demographic factors on knowledge, preparedness and counseling practice.

Variable	Knowledge score median (IQR)	*P-value	Preparedness score median (IQR)	*P-value	Practice score median (IQR)	*P-value
**Gender**						
Male	8 (7–9)	0.11	9 (7–12)	<0.0001	25 (21–29)	0.13
Female	8 (7–9)		8 (5–10)		24 (21–28)	
**Age, years**						
≤28	8 (7–9)	0.65	9 (6–11)	0.43	25 (22–29)	0.32
>28	8 (7–9)		8 (6–11)		24 (20–29)	
**Experience years**						
0–2	8 (7–9)	0.174	9 (6–11)	0.120	25 (22–29)	0.22
3–5	8 (7–9)		8.5 (6–12)		24 (21–27)	
6–8	8 (7–9)		7 (4–11)		24 (21–29)	
≥9	8 (7–9)		8 (5–11)		25 (20–29)	
**Working place**			8.5 (6–11)			
Governmental hospital	8 (7–9)	0.70	8 (6–11)	0.17	25 (21–28)	0.70
Private hospital	8 (7–9)		9 (6–11)		24 (22–29)	
Primary healthcare centre	8 (8–9)		7 (4–11)		24 (21–28)	
Missing						
**Profession**						
Physician	8 (7–9)	0.007	9 (6–12)	0.19	24 (21–29)	0.14
Nurse	8 (7–8)		8 (6–11)		25 (22–28)	
Physician assistant	8 (7–9)		9 (6–10)		26 (18–30)	
Pharmacist	8 (7–9)		8.5 (4–11)		23 (20–27)	
Physician vs Nurse		0.001				
Physician vs Physician assistant		0.09				
Physician vs Pharmacist		0.52				
Nurse vs Physician assistant		0.30				
Nurse vs Pharmacist		0.10				
Physician assistant vs Pharmacist		0.49				
**Department**						
ICU	8 (7–8)	0.10	11 (8–13)	<0.0001	24 (20–30)	0.229
Emergency	8 (7–9)		10 (7–12)		25 (22–29)	
Internal medicine	8 (7–9)		9 (7–12)		25 (23–28)	
Others	8 (7–9)		8 (5–10)		24 (21–28)	
ICU vs Emergency				0.57		
ICU vs Internal medicine				0.14		
ICU vs Others				<0.0001		
Emergency vs Internal medicine				0.30		
Emergency vs Others				<0.0001		
Internal medicine vs Others				0.005		

*Mann-Whitney U test and Kruskal–Wallis were used. IQR = Interquartile range.

### Perceived barriers to adequate prevention and control of COVID-19

**Fig 2:**
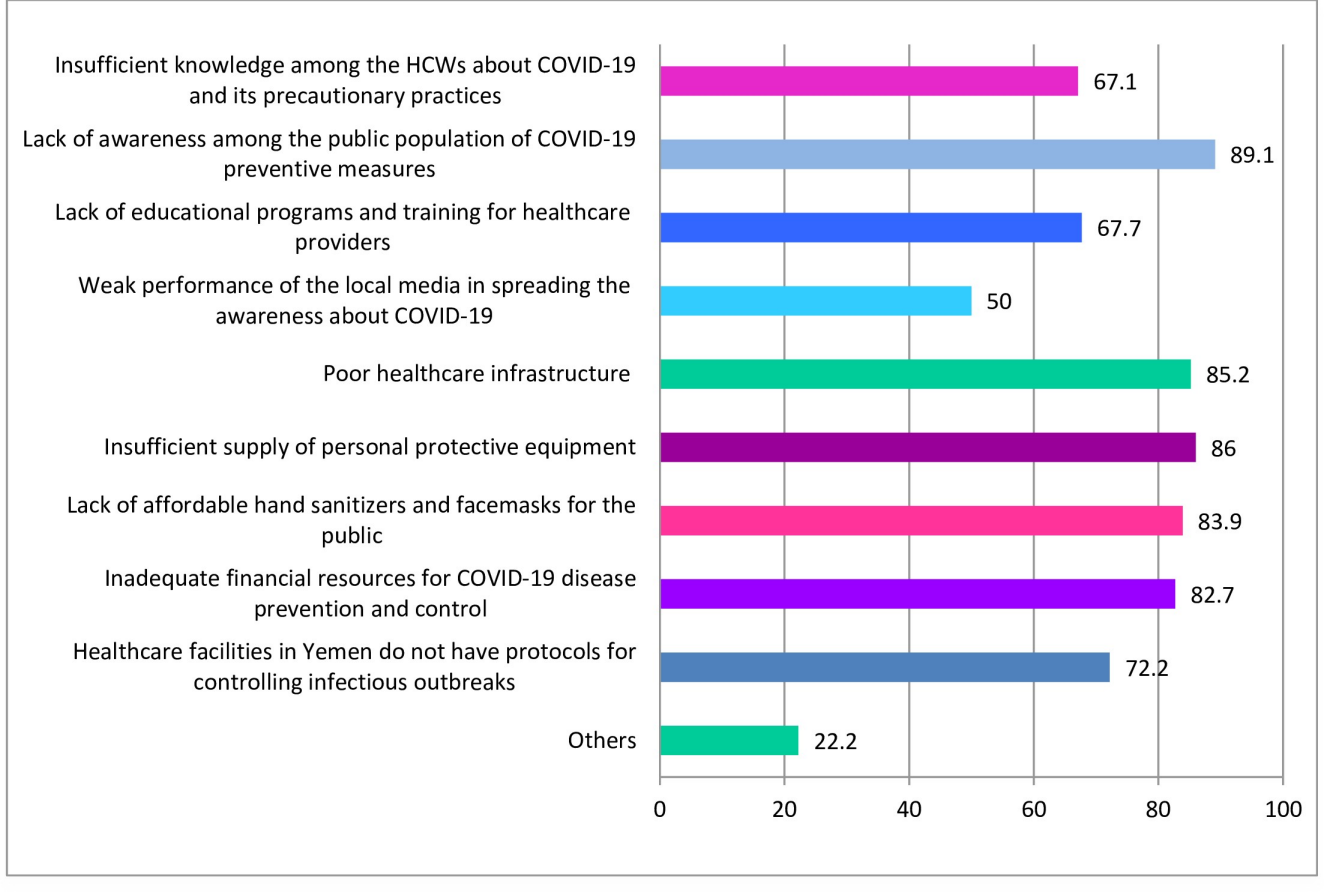
Perceived barriers to adequate prevention and control of COVID-19 in Yemen.

The participants reported a range of barriers towards the adequate prevention and control of COVID-19 in Yemen. The most common one was the lack of awareness among the general population about COVID-19 preventive measures (89.1%). Other common perceived barriers included poor healthcare infrastructure (85.2%), an insufficient supply of personal protective equipment (PPE) (86%), lack of affordable hand sanitisers and facemasks for the public (83.9%), and inadequate financial resources for COVID-19 prevention and control (82.7%). Half of the participants perceived the weak performance of the local media in spreading the awareness of COVID-19 as a barrier. The lack of training on infectious disease outbreaks and the absence of emergency response protocols were also perceived as barriers by 67.7% and 72.2% of respondents, respectively (Fig 2).

## Discussion

The findings of this study revealed that the knowledge among HCWs was adequate, with a median knowledge score of 8 out of 9 (~ 88.8%). This is consistent with recent studies that demonstrated high knowledge scores among HCWs. For example, in a study that included ten tertiary care settings from Henan province, which shares borders with Hubie province where the outbreak started, they found that around 89% of HCWs had sufficient knowledge of COVID-19 [21], which is close to the findings of this study. Also, Olum et al., 2020 reported similar results of around 82.4% of HCWs demonstrating good knowledge about COVID-19 [20]. In contrast, the previous survey by Bhagavathula et al. showed poor knowledge among HCWs [19]. Another study was conducted in Libya, a war-torn country like Yemen, reporting a low overall knowledge score (26.5%) among frontline healthcare providers [18]. Yemen has been one of the latest countries reporting COVID-19 cases since the pandemic has already spread to most countries worldwide [24]. Also, the extensive awareness coverage globally and the state of emergency worldwide of the pandemic provided HCWs in Yemen with a distinct advantage to leverage and take advantage of the information already disseminated about COVID-19. Nevertheless, almost 37% of HCWs in this study were unable to identify the correct answer when asked about the possibility of asymptomatic patients to transmit the virus. Recent studies have suggested that asymptomatic individuals could be an important factor in the transmission of SARS-CoV-2 [25–27]. Therefore, HCWs’ knowledge of such information could be of clinical significance in the process of prevention and control of COVID-19.

Interestingly, a significant difference in knowledge was observed between physicians and nurses, which could be partially explained by the fact that physicians play a more prominent role in the management and treatment of COVID-19; their position in the healthcare system mandates them to search for updated information and to answer all relevant questions of patients and the community about COVID-19. Notably, television and social media were the primary sources of information about COVID-19, which is in line with previous studies reporting similar findings [18, 19]. Therefore, these information sources could be utilised by healthcare authorities, local and international NGOs to promote awareness and providing up-to-date informational messages about COVID-19 to the community and other stakeholders.

Regarding preparedness, HCWs were not optimally prepared in dealing with COVID-19, even though most of them demonstrated good knowledge about the virus. Females were less prepared as compared to their male counterparts. The difference in preparedness could be due to self-reporting response bias between females and males. Previous research reported that female medical students and residents had less confidence in issues related to competence compared to males, and were inclined to underestimate their skills and abilities [28–30]. Therefore, in the present study, females might have answered with ‘I am not sure’ for preparedness questions more so than males, and males might have answered with ‘yes’, even if their preparedness was not 100% adequate.

Several gaps were identified in this study regarding preparedness among HCWs. In particular, almost a third of the participants reported the inability to identify the signs and symptoms of COVID-19, and more than half lacked the skills needed to manage and prioritise COVID-19 patients. Additionally, almost 50% of respondents were unsure or did not know to whom and to where COVID-19 cases should be reported. Similarly, the majority of HCWs did not participate in educational activities and emergency planning for COVID-19. Accordingly, these findings could be attributed to the absence of pre-emptive plans and emergency response protocols for COVID-19 at healthcare facilities, lack of educational activities, and insufficient professional training for COVID-19 among the participants [6]. Moreover, the lack of such essential triage skills and protocols could lead to inappropriate resource utilisation, treatment delays and overcrowding in hospitals, which could increase the risk of cross*-*contamination [31]. Therefore, COVID-19 training on triage skills is essential and should be implemented for all frontline HCWs in Yemen. The inadequate preparedness among HCWs was mentioned previously as a factor that contributed to the wide misdiagnosis of the Ebola virus disease during the West African outbreak between 2014 and 2015 [32, 33]. In the United Kingdom (UK), Prescott et al. assessed the preparedness of HCWs using a cross-sectional online survey where they moderate readiness for COVID-19 among HCWs [34]. In a more recent study in Libya, it showed low levels of awareness and preparedness among frontline physicians and nurses regarding COVID-19 [18].

The participants of this study reported many barriers towards adequate prevention and control of COVID-19 in Yemen. Lack of awareness among the general population about COVID-19 along with the absence of affordable facemasks and hand sanitisers were among the major barriers reported by HCWs. This fact is further complicated in a country like Yemen, in which 20.4 million people are in need of sanitation, safe water, and hygiene assistance, with up to 70% of Yemeni’s lacking access to soap for handwashing and personal hygiene [35]. Therefore, for adequate prevention and control of COVID-19, these barriers need to be urgently addressed by government authorities and NGOs.

Other significant barriers include poor healthcare infrastructure (85.2%) and insufficient supply of PPE (86%), which is in line with previous international reports documenting how fragile the healthcare system is in Yemen. The WHO reported that only 51% of healthcare facilities in Yemen are fully functional [36]. On July 4, 2020, the UN agencies reported that Yemen had only 380 ICU ventilators and 780 ICU beds nationwide [37]. Recently, the principals of the Inter-Agency Standing Committee expressed great concern and alarm about the situation in Yemen amid the COVID-19 crisis, where they reported the lack of basic equipment like facemasks, protective gear, gloves, and other essential supplies for COVID-19 management [13]. PPE is extremely important during any disease outbreak as HCWs are at increased risk of infection, and the presence of PPE can help to mitigate this risk [38]. Therefore, urgent attention and actions are needed to provide adequate PPE for the HCWs in Yemen.

Despite these perceived barriers, the HCWs showed good counselling practices for a country overburdened with problems and obstacles, having no more than 15.5% of inappropriate counselling practices. Before the outbreak of COVID-19, the majority of participants in this study educated their patients and the community about the measures to prevent and control COVID-19, such as social distancing, avoiding public gatherings, and regularly washing their hands before touching their face and eyes. Their roles became more prominent following the outbreak of COVID-19 as they continued to work during this crisis, risking their lives to save their patients. According to a recent international report, 97 HCWs have died from COVID-19 in Yemen [5]. Furthermore, given the repeated infectious outbreaks such as cholera, diphtheria, and dengue, and considering the lack of PPE, with no incentives or salaries paid to HCWs [13], the remaining healthcare workforce in Yemen are stressed and facing burnout.

In Yemen, several awareness campaigns and initiatives were undertaken by Yemeni civil society organisations, and international organisations to promote public awareness about COVID-19 and preventive measures [13, 39]. Various interventions have also been implemented by international organisations (the WHO and UN agencies) to promote the preparedness and response of the healthcare system and HCWs in managing COVID-19 in Yemen [13, 37]. However, these efforts are lagging with a number of gaps still present. Accordingly, all HCWs from different disciplines must be trained in the prevention, control, and managing COVID-19. This also includes the need to be well prepared and appropriately protected, which is crucial in reducing the spread of the disease, and preparing for worst-case scenarios. Data from a recent modelling projection for COVID19 in Yemen predicts that as many as 10 million people could become infected with Covid-19, with up to 85,000 deaths in the worst-case scenario, if no interventions are implemented [40]. This could be devastating not only for the healthcare system but also for HCWs. Previous studies conducted in Yemen have shown that training of healthcare providers during disasters was associated with improving their skills [41] and increasing their willingness to participate in a disaster [42]. Therefore, training, providing up-to-date knowledge, resources and protecting HCWs is urgently required in preparing them for the worst-case scenario of the COVID-19 outbreak in Yemen.

### Strengths and limitations

There are several limitations inherent in this study. One limitation is that the study was only undertaken in Sana’a, the capital of Yemen, and not all healthcare facilities participated in the study, which limits the generalisability of the findings. Also, selection bias cannot be excluded as convenience sampling was employed in selecting the participants. Moreover, the possibility of response bias may prevail as the participants might have given socially desirable responses for questions related to self-preparedness and counselling practices. Furthermore, as a cross-sectional study, the results represent only a point-in-time in which the data were collected and does not consider any change in preparedness over time. Another limitation is related to the questionnaire. Due to time constraints and the urgency in distributing the survey to the participants, it was only face validated by four independent healthcare professionals. Thus, a more in-depth evaluation of the questionnaire’s validity and reliability may have produced a more robust tool.

Nevertheless, despite these limitations, the sample size in the present study was considered adequate with most of the major private and governmental hospitals in Sana’a included. The study also provides a baseline level of knowledge and preparedness among HCWs, allowing for future comparative work and intervention progress assessment to be undertaken in future. As such, this study provides a clear picture of the barriers and limitations of the healthcare system in Yemen regarding the COVID-19 pandemic. Accordingly, the results of this study could be utilised by local healthcare authorities and international health organisations in designing and delivering appropriate interventions for HCWs in Yemen to enhance their knowledge, practice, and preparedness for the COVID-19 crisis or for any future infectious disease outbreaks.

## Conclusion

The major highlight of this study is that HCWs were noted to have overall, good knowledge, suboptimal preparedness, and adequate counselling practices before the outbreak of COVID-19 in Yemen, despite the high number of perceived barriers. Urgent actions and interventions are needed to improve the level of preparedness of the HCWs to manage the spread and containment of COVID-19. The perceived barriers need to be acted upon and addressed by local healthcare authorities and international organisations working in Yemen for adequate prevention and control of the COVID-19.

Based on the findings of this study, the following recommendations are presented:

- All frontline HCWs should be trained on the measures to prevent, control, and manage COVID-19. This is to enhance their knowledge, improve their preparedness and practice to the desired level to cope with the crisis.
- HCWs need to be appropriately protected and providing them with adequate PPE is fundamental.
- Healthcare authorities should develop protocols for COVID-19 prevention, control and management specific to the country’s current challenges and context.
- The international community and local authorities are urged to provide adequate medical supplies that are essential to combat COVID-19, which could be actualized through the realisation of makeshift hospitals, since the fragile healthcare system of Yemen is ill-equipped to deal with the SARS-CoV-2 outbreak.
- Local authorities should provide free and affordable hand sanitiser(s) and facemasks to low-income households. Also, implementing massive awareness campaigns and other activities regarding COVID-19 is required. These measures are crucial for adequate control and prevention of COVID-19 in Yemen.

## Supporting information

S1 File Study questionnaire. (PDF)
