# Supplementary material for: Healthcare workers’ knowledge, preparedness, counselling practices, and perceived barriers to confront COVID-19: A cross-sectional study from a war-torn country, Yemen
Source: PLoS One. 2020 Dec 11;15(12):e0243962. doi: 10.1371/journal.pone.0243962 (PMC7732096; doi:10.1371/journal.pone.0243962)
Supplement: S1 File — (PDF) [file pone.0243962.s001.pdf]

Knowledge, preparedness, counselling practices, and perceived barriers among healthcare workers regarding the COVID-19

Section one: Demographic characteristics

**Age**	……………..	
**Gender**	☐ Male<br>☐ Female	
**Profession**	☐ Physician ☐ Pharmacist ☐ Nurse ☐ Physician- assistant	
**Department**	☐ ICU ☐Emergency ☐ Internal medicine<br>☐ Other departments (Please specify):	
**Place of current work**	☐ Private hospital ☐ Public hospital<br>☐ Primary healthcare centre	
**1-Number of experience years**	………………….	
**2-Did you attend any Workshops or courses related to the COVID-19 outbreak?**	☐Yes	☐ No
**3-Have you ever been trained on control and prevention of COVID-19 outbreak?**	☐Yes	☐ No
**4-Have you ever been trained on control and prevention of infectious diseases outbreak?**	☐Yes	☐ No
**5-Do you have previous experience in managing infectious disease outbreaks?**	☐Yes	☐ No
**6-What is/are the main source/s of information for your knowledge about COVID-19 disease?**		
☐ Media (e.g. TV, radio, Newspaper)<br>☐ Your healthcare colleagues<br>☐ Social applications and websites (Facebook, WhatsApp, Telegram and other Social apps)<br>☐ Trusted medical websites (e.g., the World Health Organization, Medscape,…)<br>☐ Scientific journals<br>☐ Others (please specify):……………………		

Section two: Healthcare professionals' knowledge of COVID-19

1	COVID-19 is an infectious disease caused by a virus called severe acute respiratory syndrome coronavirus 2 (SARS-CoV-2)	☐Yes	☐No	☐ I don't know
2	The most common symptoms of COVID-19 are fever, tiredness, shortness of breath and dry cough	☐Yes	☐No	☐ I don't know
3	Older people, and those with underlying medical problems like heart problems or diabetes, are more likely to develop serious illness	☐Yes	☐No	☐ I don't know
4	The disease spread from person to person through small droplets from the nose or mouth when a person with COVID-19 coughs, sneezes or exhales	☐Yes	☐No	☐ I don't know
5	There is a chance that people may get infected from asymptomatic COVID-19 patient	☐Yes	☐No	☐ I don't know
6	Studies indicate that the virus causing COVID-19 may remain alive on surfaces for a few hours to several days	☐Yes	☐No	☐ I don't know
7	The time between catching the virus and beginning to have symptoms of the disease (incubation period) for COVID-19 range from 1-14 days	☐Yes	☐No	☐ I don't know
8	The use of masks is crucial for health workers and people who are taking care of someone (at home or in a health care facility)	☐Yes	☐No	☐ I don't know
9	Currently, there is no vaccine to prevent COVID-19 disease	☐Yes	☐No	☐ I don't know

Section three: Healthcare professionals' preparedness for COVID-19 outbreak

1	I know all the relevant information related to COVID-19 disease.	☐Yes	☐No	☐ Not sure
2	I have read general guidance about COVID-19 (for example, WHO or CDC COVID-19 guidance).	☐Yes	☐No	☐ Not sure
3	I have read medical articles related to COVID-19 outbreak preparedness.	☐Yes	☐No	☐ Not sure
4	I can identify the signs and symptoms of COVID-19.	☐Yes	☐No	☐ Not sure
5	I can manage the common symptoms of COVID-19.	☐Yes	☐No	☐ Not sure
6	I have the skills to decide which patients should be managed first.	☐Yes	☐No	☐ Not sure
7	I can care for COVID-19 patients independently without any supervision.	☐Yes	☐No	☐ Not sure

8	I have participated in educational activities dealing with COVID-19 outbreak preparedness (continuing education classes, seminars, or courses).	☐Yes	☐No	☐ Not sure
9	I have the necessary knowledge and skills to educate patients about COVID-19 prevention practices.	☐Yes	☐No	☐ Not sure
10	I am aware of all the challenges in my community that may hinder the response to the COVID-19	☐Yes	☐No	☐ Not sure
11	I know how and to whom COVID-19 cases should be reported.	☐Yes	☐No	☐ Not sure
12	I have participated in emergency planning for COVID-19 in my community	☐Yes	☐No	☐ Not sure
13	In case of infectious disease outbreaks, I know how to use properly the personal protective equipment	☐Yes	☐No	☐ Not sure
14	In case of infectious disease outbreaks, I know how to execute decontamination procedures.	☐Yes	☐No	☐ Not sure
15	In case of emergency, I know how to perform isolation procedures to minimize the risks of community exposure.	☐Yes	☐No	☐ Not sure

Section four: Healthcare professionals' practices of counselling patients regarding COVID-19 prevention measures

		Never	Rarely	Sometimes	Usually	Always
**1**	Do you educate the patients and the people around you about the risk of COVID-19?					
**2**	Do you educate the patients and the people around you about the symptoms, signs, and mode of transmission of COVID-19?					
**3**	Do you educate the patients and the people around you about the preventative measures of COVID-19?					

**4**	Do you advise the patients and the people around you to avoid public gatherings (such as khat sessions, weddings, malls, markets, and restaurants)					
**5**	Do you educate the patients and the people around you about the importance of handwashing before touching their eyes, nose, or mouth?					
**6**	Do you educate the patients and the people around you on what to do if they developed similar symptoms and signs to COVID-19 disease					

Section five: Perceived barriers for adequate COVID-19 prevention and control in Yemen

In this section, please select all the factors that you think as a healthcare worker could lead to improper control of the disease in Yemen (please choose all that apply):

☐ Insufficient knowledge among healthcare providers regarding the COVID-19 and its precautionary practices
☐ Lack of awareness among the public population of the preventive measures for COVID-19
☐ Lack of educational programs and training for healthcare providers on infectious diseases outbreaks
☐ Weak performance of the local media in spreading the awareness about COVID-19 pandemic
☐ Poor healthcare infrastructure
☐ Insufficient supply of personal protective equipment (PPE)
☐ Lack of affordable hand sanitizers and facemasks for the public
☐ Inadequate financial resources for COVID-19 disease prevention and control
☐ Healthcare facilities in Yemen do not have protocols for controlling infectious outbreaks

☐ Others, if you have other barriers for adequate control of COVID-19 in Yemen, please write them here:
……………………………………………………………………………………………
……………………………………………………………………………………………
……………………………………………………………………………………………
